# Generalizable machine learning approach for COVID-19 mortality risk prediction using on-admission clinical and laboratory features

**DOI:** 10.1038/s41598-023-28943-z

**Published:** 2023-02-10

**Authors:** Siavash Shirzadeh Barough, Seyed Amir Ahmad Safavi-Naini, Fatemeh Siavoshi, Atena Tamimi, Saba Ilkhani, Setareh Akbari, Sadaf Ezzati, Hamidreza Hatamabadi, Mohamad Amin Pourhoseingholi

**Affiliations:** 1 Basic and Molecular Epidemiology of Gastrointestinal Disorders Research Center, Research Institute for Gastroenterology and Liver Diseases, Shahid Beheshti University of Medical Sciences, Tehran, Iran; 2 Department of Surgery, Center for Surgery and Public Health, Brigham and Women’s Hospital, Harvard Medical School and Harvard T.H Chan School of Public Health, Boston, MA USA; 3 Department of Emergency Medicine, School of Medicine, Safety Promotion and Injury Prevention Research Center, Imam Hossein Hospital, Shahid Beheshti University of Medical Sciences, Tehran, Iran

**Keywords:** Medical research, Risk factors, Microbiology, SARS-CoV-2

## Abstract

We aimed to propose a mortality risk prediction model using on-admission clinical and laboratory predictors. We used a dataset of confirmed COVID-19 patients admitted to three general hospitals in Tehran. Clinical and laboratory values were gathered on admission. Six different machine learning models and two feature selection methods were used to assess the risk of in-hospital mortality. The proposed model was selected using the area under the receiver operator curve (AUC). Furthermore, a dataset from an additional hospital was used for external validation. 5320 hospitalized COVID-19 patients were enrolled in the study, with a mortality rate of 17.24% (N = 917). Among 82 features, ten laboratories and 27 clinical features were selected by LASSO. All methods showed acceptable performance (AUC > 80%), except for K-nearest neighbor. Our proposed deep neural network on features selected by LASSO showed AUC scores of 83.4% and 82.8% in internal and external validation, respectively. Furthermore, our imputer worked efficiently when two out of ten laboratory parameters were missing (AUC = 81.8%). We worked intimately with healthcare professionals to provide a tool that can solve real-world needs. Our model confirmed the potential of machine learning methods for use in clinical practice as a decision-support system.

## Introduction

As of 25 September 2022, 612 million confirmed cases and 6.5 million deaths due to COVID-19 have been reported globally (WHO, 2022)^1^. Even after vaccination, the peaks in the incidence of COVID-19 have arisen as new variants challenge former immunization^2^. Assessing the risk of COVID-19 fatality can guide clinical decision-making by healthcare professionals^3^. Many studies have investigated the predictors of COVID-19 death and severity and proposed risk stratification tools^4^.

Machine learning (ML), as a novel approach, can improve policy-making, forecasting, screening, drug development, and risk stratification. Artificial intelligence (AI) can result in fair decision-making by minimizing interobserver variability and filling the gap between healthcare resources and human workload^5^. Although many ML algorithms have strived to help physicians, ML tools face several obstacles to implementation in clinical practice. For instance, the clinicians' hardship in using and interpreting computational models may hinder the further progress of ML. Thereby, creating a reproducible easy-to-use model is vital, which can be achieved with healthcare professionals’ assistance in model development. Moreover, training a generalizable ML needs precise data collection and population selection. In this fashion, the ML training data set will represent the actual population using the model in the future^6^.

Risk stratification of patients can indicate the most vulnerable groups and is crucial for resource allocation and follow-up of patients^5^. Table 1 summarizes previous studies on the prediction of COVID-19 mortality. A systematic review of prediction models for COVID-19 mortality showed that 70 out of 79 articles faced a high or unclear risk of bias^7^. Even among the nine articles with a low risk of bias, external validation was not considered in six^7^. Therefore, the reproducibility of ML experiments on this matter can be in question. In addition, collecting a large set of predictors is time-consuming, and many studies with a large number of clinical and laboratory predictors tend to have a limited patient population (Table 1). On the other hand, reducing the number of collected features may compromise a precise interpretation of the disease and its severity since COVID-19 is a multi-organ disease^8^.

**Table 1:** Studies with or without external validation aiming to predict prognosis of COVID-19 using clinical and laboratory features (retrieved from review articles and search in PubMed and Scopus databases^7,9^).

Author, publish date,	Training dataset sources, country	Number of patients for model development	Variable for prediction	Outcome	Proposed model	Internal** (In) and external (Ex) validation AUROC (95% CI)
Our model	3 centers, Iran	5320	27 clinical (history and examination) and 10 laboratory variables	In-hospital mortality	Deep neural network, LASSO	In: 83.8% Ex: 82.8%
Studies with external validation						
Singh et al. 2021^10^	3 centers,	8,427	10 markers selected from 57 laboratory, clinical, and demographic variables	Disease severity*	minimum redundance maximum relevance, hybrid feature selection	In: 78% Ex: 74%
Noy et al. 2022^11^	1 center, Israel	417	Static and dynamic features including demographics, background disease, vital signs and lab measurements	deterioration within the next 7–30 h	CatBoost (ensemble decision tree)	In: 84% Ex: 74%
Chen et al. 2021^12^	7 centers, China	6415	4 Clinical and 4 Laboratory Variables	In-hospital mortality	Random forest, LASSO	In: 90% Ex: 89%, 90%, 81%
Clift et al. Oct 2020^13^	910 practices, UK	6,083,102	age, ethnicity, deprivation, body mass index, and a range of comorbidities	In-hospital mortality	regression coefficients, LASSO	AUROC is not reported, R squared = 73.1%
Vaid et al. 2020^14^	1 center, USA	1514	Age and 8 laboratory markers	In-hospital mortality (following 1,3,5,7 days)	XGBoost, LASSO	In: 89% at 3 days, 85% at 5 and 7 days Ex: 80% at 3 days, 79% at 5 days, 80% at 7 days
Ko et al. 2020^15^	1 center, China	361	Age, gender, and 28 blood biomarkers	In-hospital mortality	deep neural network and random forest models	In: accuracy = 93% Ex: accuracy = 92%
Gao et al. 2020^16^	2 centers, China	1506	6 clinical and 2 laboratory biomarkers	mortality risk stratification	Logistic regression, support vector machine, gradient boosted decision tree, and neural network	In: 92.4%, Ex: 95.5%, 87.9%
Bertsimas et al. 2020^17^	33 centers	3,927	Age and 9 laboratory biomarkers	In-hospital mortality	XGBoost	In: 90% Ex: 87%, 92%, 80%
Guan et al. 2021^18^	2 centers, China	1270	2 clinical and 4 laboratory features	In-hospital mortality	Simple-tree XGBoost	In:99.1% Ex: 99.7%
Hu et al. 2020^19^	1 center, China	183	Age and 4 laboratory variables	In-hospital mortality	Logistic regression	int:89.5% Ex: 88.1%
Studies without external validation						
Shanbehzadeh et al. 2022^20^	1 center, Iran	1710	13 from 58 features selected including 5 symptom, 4 laboratory, pleural fluid, ICU admission, LOS, age	In-hospital mortality	ANN, back propagation	Int: 85.3% Ex: –
Napour et al. 2022^21^	1 center, Iran	482	ICU admit, LOS, 3 laboratory, underlying disease, 7 clinical, oxygen therapy,	In-hospital mortality	ANN	Int: 90% Ex: –
Das et al. 2020^22^	CDC, Korea	3,524	Age, gender, province, exposure	Mortality (community risk)	Logistic regression with SMOTE	Int: 0.83 Ex: –
Goodacre et al. 2021^23^	70 centers, UK	20,889	Age, sex, 5 vital signs, performance status, consciousness	Mortality, organ support*** in 30 days	LASSO	In: 80% Ex: –
Knight et al. 2020^24^	260 centers UK	35 463	Age, sex, number of comorbidities, RR, O2 sat, consciousness, 2 laboratories	Mortality risk	XGBoost, GAM, LASSO	In: 77% Ex: –
Lopez-Escobar et al. 2021^25^	10 centers, Spain	1955	Age, sex, O2 sat, 4 laboratories	In-hospital mortality	Logistic regression	In: 86% Ex: –
Wollensteid-Betech et al. 2020^26^	All COVID-19 cases, Mexico	91,000	Age, sex, 8 comorbidities, COVID-19 test result, tobacco use	Mortality, hospitalization, ICU need, ventilator need	Logistic regression, SVM	In: 72%, 79%, 89%, and 90% for mortality, hospitalization, ICU need, and ventilator need Ex: –

*LOS* length of stay, *ICU* intensive care unit, *AUROC* area under the receiver operating characteristic, *LASSO* least absolute shrinkage and selection operator, *ANN* artificial neural network, *SMOTE* synthetic minor oversampling technique, *RR* respiratory rate, *SBP* systolic blood pressure, *GAM* generalized additive model.

*Severity level 0 (no respiratory problem) to level 4 (in-hospital ≤ 30-day mortality).

**For internal validation the evaluation metrics on test model was retrieved.

***Organ support assumed as need for respiratory, renal, or cardiovascular support.

This study aims to propose an on-admission mortality risk prediction model and investigate its external validation to assess the generalization of the tool. In order to increase the ease of implementation, we gather feedback from clinicians involved in COVID-19 practice. This study is part of an observational, retrospective, multicentric research project to investigate the epidemiological characteristics of COVID-19 patients^27^.

## Material and methods

### Data collection

We used data set of 5320 confirmed COVID-19 patients admitted to three general hospitals in Tehran, Iran, from March 2020 to March 2021. A Medical team reviewed patients' medical records and gathered patients' demographics, symptoms, comorbidities, admission vital signs, and outcomes. Laboratory results were collected for all patients on the first day of admission through the hospital information system. Confirmation of cases was based on real-time polymerase chain reaction (RT-PCR) for SARS-CoV-2 of nasal or oropharyngeal swab samples on the first days of hospitalization. The outcome of current study was death versus discharge from the hospital. We previously explored the epidemiology of the cohort used in this study in detail^27^.

### Data cleaning and imputation

Patients with any missing categorical variable or missing more than two numerical features were removed from the dataset. Out of 88 features collected from cohort patients, including 52 categorical features and 29 continuous features, none of the categorical features contained missing data. Conversely, seven numerical features were dropped due to a proportion of missing values greater than 5%. Other missing values were imputed using Python's Sci-kit learn iterative imputer.

### Feature selection

Feature selection can prevent overfitting, a sinficant problem in ML models, by eliminating redundant collinear features. We recognized the most predictive values using the least absolute shrinkage and selection operator (LASSO) regression and Boruta feature selection methods. LASSO confirmed 37 features containing 25 categorical and 12 nominal features, and Boruta selected 24 features, all of which were nominal. We used these groups separately as our training data features and compared the performances.

### Model development

**Figure 1:**
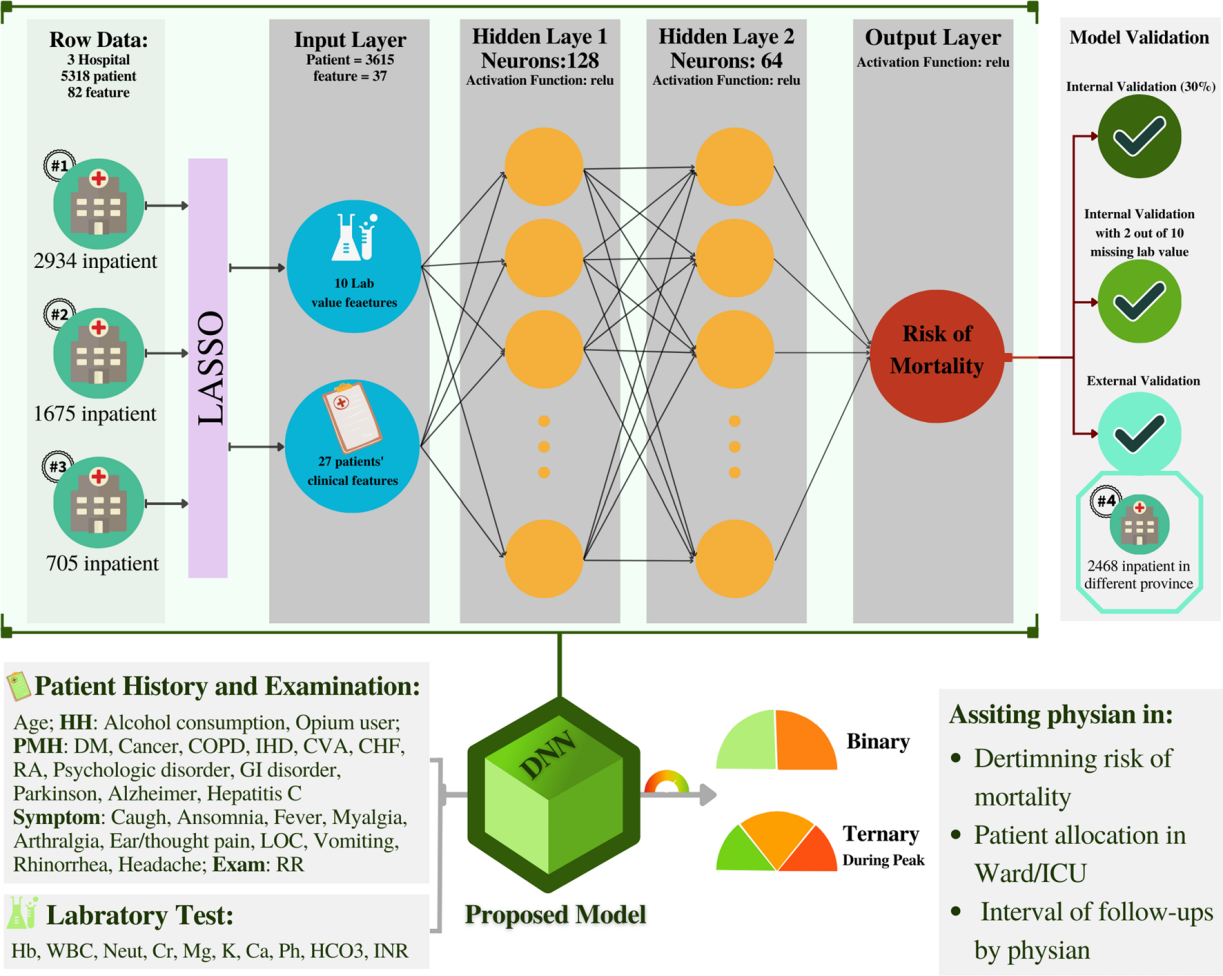
Proposed deep neural network model structure and implementation (*LASSO* least absolute shrinkage and selection operator, *DM* diabetes, *COPD* chronic obstructive pulmonary disease, *IHD* ischemic heart disease, *CVA* cerebrovascular accident, *CHF* chronic heart failure, *RA* rheumatoid arthritis, *GI* gastrointestinal, *LOC* loss of consciousness, *RR* respiratory rate, *Hb* hemoglobin, *WBC* white blood cell, *Neut* neutrophil count, *Cr* creatinine, *Mg* magnesium, *K* potassium, *INR* international normalization ratio of prothrombin time, *DNNL* deep neural network, *ICUL* intensive care unit).

Six ML classification models were trained and fine-tuned, including support vector machine (SVM) with Radial Basis Function (RBF) as kernel and the degree set to 3, logistic regression (LR), k-nearest neighbors (KNN) with number of neighbors set to 5 and weights to uniform, random forest (RF) with the number of estimators set to 100 and criterion set to Gini, gradient boosting decision tree (GBDT) with the number of estimators set to 100, learning rate set to 0.1, and loss set to log_loss, and deep neural network (DNN) to calculate the risk of mortality in admitted covid patients. SVM and LR were regularized using the L2-regularization (Ridge regression) method. After fine-tuning, the neural network contained two hidden layers with 128 and 64 units for the first and second hidden layers, respectively. Moreover, all layers were activated using rectified linear unit (ReLU) activation function, and the output layer contained a unit with a sigmoid activation function. All layers except the output layer had 60% dropout. A DNN compiled with binary cross-entropy as loss function and stochastic gradient descent with learning rate, decay, momentum, and Nesterov set to 0.01, 1e−7, 0.9, and true as optimizer, respectively. The ML pipeline of the proposed DNN model and its implementation are depicted in Fig. 1.

### Model training and evaluation

Two data sets were created using features confirmed by each feature selection method. Then datasets were randomly split into training and validation sets in a ratio of 7:3 while preserving the same proportion of mortality in all datasets due to the small percentage of mortality in datasets.

Using accuracy for evaluating model performance was inappropriate due to the skewness of the data. Precision, recall, F1-Score, sensitivity, specificity, and area under the curve (AUC) of the receiver operating characteristic (ROC) score were calculated to evaluate model performance on validation datasets. Additionally, the ROC curve visualized model performance.

After each iteration of model training and validation, we fine-tuned model parameters, including the number of layers, number of neurons in each layer, learning rate, regularization method, and perceptron connection dropout rate for the ANN models. Also, we tuned parameters like the number of estimators for gradient boosted classifier, the maximum depth for the RF model, and the regularization method for SVM and LR models. These fine-parameter changes were used to maximize the accuracy and generalizability of our AI models. Finally, we tested our trained models' performances on an external dataset from another tertiary hospital in a different province of Iran to evaluate the generalizability of our models.

### Effect of using iterative imputer on models' performances

One of the most critical issues that every ML and deep learning project on tabular data must overcome is dealing with missing data. There are several ways to solve this problem, including filling with median, mean, arbitrary value, previous/next value, using the most common value, and imputing the missing values using ML models. In this study, we used an iterative multivariate imputer, which estimates the missing values in each feature using all other features in the dataset. This is one of the most commonly used ML strategies for missing values. We evaluated the effect of the iterative imputer on ML models' performances and compared it with models trained on datasets without missing values. For this comparison, we randomly removed 20% of the numerical values in our training datasets and trained the same ML models with the same hyperparameters on these datasets. Then we evaluated the performance metrics of these models on the primary testing dataset to compare their performances.

### Optimal cutoff point

Expert opinions of an emergency medicine professor, an internist, and two general practitioners were collected on optimal cutoff points of the proposed model. Two systems with binary (high risk, low risk) and ternary (very high risk, high risk, low risk) classifications were suggested. The ternary classifications can help physicians during peaks of the disease to find the most susceptible patients and allocate hospital beds properly. The optimal cutoff scores were selected based on the optimal point of ROC and the clinician's opinion after reviewing the probability graph. A confusion matrix was used to visualize the performance of cutoff scores in a randomly selected sample from the external validation dataset with 100 survived and 100 deceased cases.

### Statistical analysis

Data analysis and visualization were performed using the R program. The Kolmogorov–Smirnov normality test was used to evaluate the normal distribution of a variable. The Fisher exact test was used to determine the significance of categorical features, and the Mann–Whitney U test was used to evaluate the significance difference of non-parametric numerical variables. An Independent t-test was used to find the significant difference in parametric numerical features. Cox proportional hazards model was used to find the odds ratio (OR) of time-to-death. The categorical variables are presented as numbers and percentage, and numerical variables are presented as mean and standard deviation (SD).

### Ethical approval

All methods were performed in accordance to Helsinki protocol. The Institutional Review Board (IRB) at the Shahid Beheshti University of Medical Science approved the study and waived informed consent gathering (IR.SBMU.RIGLD.REC.1400.014). Data were anonymized before analysis, and patient confidentiality and data security were concerned.

## Results

### Basic characteristics

After excluding 1703 patients due to missing categorical variables or missing more than two nominal variables, 5320 hospitalized COVID-19 patients were enrolled in the study with a mean ± SD age of 61.6 ± 17.6 years. The fatality rate in the enrolled cohort was 17.24% (N = 917). Patients who died due to covid-19 were significantly older than those who survived (70.3 ± 15.1 versus 58.6 ± 17.1, P < 0.001). The basic characteristics of survived and mortality cohort is presented in Supplementary Table S1.

### Factors associated with mortality

As depicted in Supplementary Table S2, on-admission factors associated with mortality in cox proportional hazards model were age, history of myalgia, loss of consciousness, vertigo and vomiting, skin lesions, alcohol consumption, history of gastrointestinal problems, rheumatoid arthritis, Neurologic disorders, leukocytosis, thrombocytopenia, low hemoglobin level, high CRP, low HCO_3_, high CPK level, low oxygen saturation, pulse rate, and respiratory rate. The most important features associated with mortality were alcohol consumption (OR 2.6) and loss of consciousness (OR 1.5). Table 2 shows the mean difference and hazard ratio of selected features.

**Table 2:** Mean comparison and Cox regression of selected variables for inclusion in the model.

Feature	Cox regression				Mean comparison*		
	HR	Lower 95% CI	Upper 95% CI	P-value	Mortality cohort	Survived cohort	P-value
Demographic and habitual history							
Age	1.028	1.023	1.034	0.001	74.00 (61.00,83.00)	60.00 (47.00,71.00)	0.001
Opium	0.827	0.581	1.178	0.293	43.0 (4.69%)	135.0 (1.06%)	0.39
Alcohol consumption	2.599	1.235	5.469	0.012	10.0 (1.09%)	11.0 (0.09%)	0.022
Comorbidities							
DM	1.09	0.936	1.27	0.266	346.0 (37.73%)	784.0 (6.17%)	0.001
IHD	1.101	0.927	1.309	0.272	214.0 (23.34%)	394.0 (3.10%)	0.001
Cancer	1.253	0.966	1.626	0.089	78.0 (8.51%)	128.0 (1.01%)	0.001
CHF	1.129	0.761	1.675	0.546	31.0 (3.38%)	52.0 (0.41%)	0.01
COPD	1.181	0.755	1.849	0.466	22.0 (2.40%)	47.0 (0.37%)	0.133
CVA	1.207	0.957	1.522	0.112	101.0 (11.01%)	134.0 (1.06%)	0.001
GI problems	1.797	1.037	3.113	0.037	15.0 (1.64%)	35.0 (0.28%)	0.271
Hepatitis C	1.348	0.185	9.805	0.768	1.0 (0.11%)	4.0 (0.03%)	0.625
Alzheimer	1.038	0.776	1.387	0.802	63.0 (6.87%)	48.0 (0.38%)	0.001
Psychological problems	1.636	1.073	2.495	0.022	24.0 (2.62%)	39.0 (0.31%)	0.017
Parkinson	1.106	0.72	1.7	0.645	25.0 (2.73%)	24.0 (0.19%)	0.001
Medical exam and history							
Respiratory rate (/min)	1.009	1.002	1.016	0.016	19 (18.00,22.00)	18 (18.00,20.00)	0.001
Fever	0.936	0.774	1.133	0.5	343 (37.40%)	1312 (10.33%)	0.001
Sore throat	0.828	0.481	1.426	0.496	14 (1.53%)	73 (0.57%)	0.046
Headache	0.881	0.668	1.164	0.374	58 (6.32%)	379 (2.98%)	0.001
Vomiting	0.83	0.696	0.99	0.038	180 (19.63%)	767 (6.04%)	0.001
Myalgia	0.825	0.688	0.988	0.037	181 (19.74%)	895 (7.05%)	0.001
Cough	0.946	0.811	1.104	0.481	373 (40.68%)	1402 (11.04%)	0.001
Arthralgia	0.992	0.555	1.775	0.979	14 (1.53%)	40 (0.32%)	0.515
Insomnia	0.925	0.38	2.253	0.864	5 (0.55%)	54.0 (0.43%)	0.001
Loss of consciousness	1.499	1.253	1.794	0.001	233 (25.41%)	179.0 (1.41%)	0.001
Rhinorrhea	1.892	0.926	3.868	0.08	9 (0.98%)	20.0 (0.16%)	0.303
Laboratory values							
Ph (VBG)	0.651	0.413	1.024	0.063	7.36 (7.29,7.41)	7.38 (7.34,7.42)	0.001
HCo3 (VBG)	0.971	0.957	0.986	0.001	23.70 (20.20,27.40)	26.00 (23.20,28.70)	0.001
Calcium	0.979	0.919	1.042	0.501	8.50 (8.00,9.10)	8.70 (8.20,9.23)	0.001
Hemoglobin (CBC)	0.962	0.931	0.995	0.025	11.80 (10.00,13.30)	12.40 (11.00,13.60)	0.001
White blood cell (CBC)	1.008	1.002	1.015	0.015	9.20 (6.30,13.30)	6.80 (4.90,9.70)	0.001
Neutrophil (%) (CBC)	1.019	1.003	1.036	0.019	85.00 (78.00,90.00)	80.00 (70.00,85.00)	0.001
INR	1.1	0.954	1.267	0.188	1.14 (1.00,1.30)	1.07 (1.00,1.20)	0.001
Potassium	1.04	0.991	1.091	0.111	4.20 (3.80,4.60)	4.00 (3.80,4.40)	0.0001
Creatinine	1.041	1	1.085	0.051	1.40 (1.10,2.20)	1.10 (0.90,1.40)	0.001
Magnesium	1.02	0.836	1.243	0.848	2.00 (1.80,2.20)	1.90 (1.80,2.10)	0.001

*VBG* venous blood gas, *DM* diabetic mellites, *INR* international normalized ratio, *CBC* complete blood count, *IHD* ischemic heart disease, *CHF* chronic heart failure, *COPD* chronic obstructive pulmonary disease, *CVA* cerebrovascular accident.

*Mann–Whitney U test was performed for evaluating difference in mean values.

### Feature selection methods and variable importance

LASSO and Brouta feature selection methods were used for variable importance, and results are visualized in Supplementary Figures S1 and S2. Twenty-four features out of 81 were confirmed by the Boruta method, mainly consisting of laboratory tests (Supplementary Figure S1). The most important features are oxygen saturation at admission, age, neutrophil count, serum level of creatinine, troponin, and loss of consciousness. Thirty-seven features were confirmed by the LASSO regression method, including 25 categorical features and 12 continuous variables (Supplementary Figure S2). Among these, 23 features were positively associated with mortality, and 14 were negatively correlated with covid patients' mortality.

### Internal and external validation

**Figure 2:**
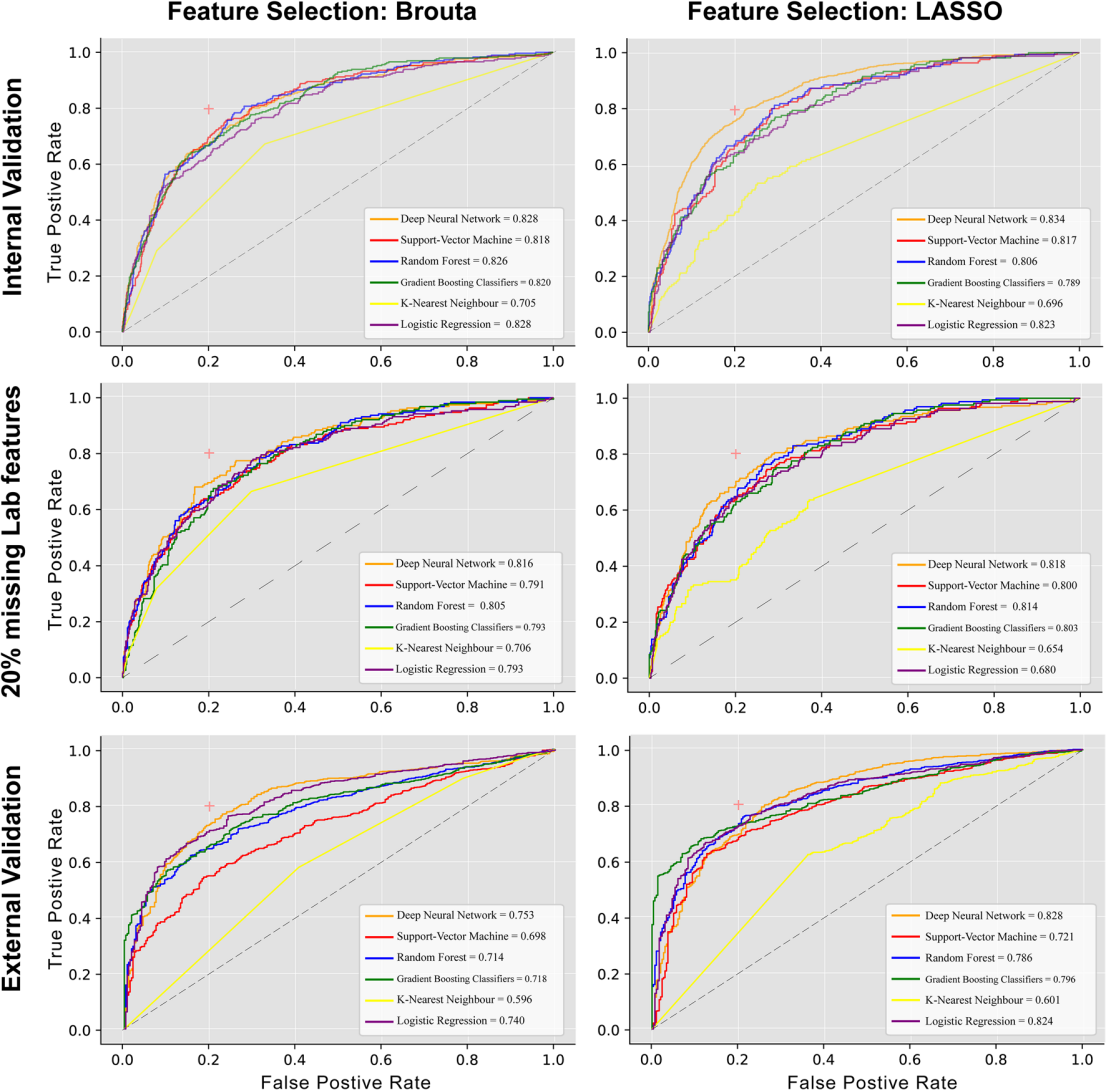
Receiver operator curve of models using two different feature selection.

The details of the model's performance in the test datasets are summarized in Table 3, and Fig. 2 shows the ROC curve of the models. Most of the trained models showed promising performance for internal validation (AUC score > 80%) except KNN, which had the lowest AUC score among all selected models in both datasets. DNN showed the best performance, with an AUC score of 83.4% in the LASSO-selected validation dataset and 82.6% in the Boruta dataset.

**Table 3:** Model internal and external validation; and validation of imputer model for 2 out of 10 missing lab value.

Feature selection method	Model	AUC score	Sensitivity	Specificity	PPV	NPV
Internal validation						
LASSO regression	DNN	83.4	62.2	92.2	70.2	89.2
	SVM	81.6	40.6	93.9	66.3	84.2
	RF	80.6	66.6	81.8	52.1	89.2
	GBDT	78.9	58.1	83.8	51.6	87.1
	KNN	69.6	31.5	88.3	44.4	81.3
	LR	82.3	44.2	90.1	57.0	84.5
Boruta	DNN	82.7	51.2	88.0	59.2	84.1
	SVM	81.7	42.1	90.1	59.1	82.1
	RF	82.5	43.2	91.6	63.6	82.6
	GBDT	82.0	44.0	90.1	60.1	82.5
	KNN	70.5	38.18	89.5	55.2	81.0
	LR	82.7	41.09	90.7	60.1	81.9
Imputer validation (two out of ten missing lab values)						
LASSO regression	DNN	81.8	60.6	86	72	79.2
	SVM	80	37.6	93.4	62.6	83.4
	RF	81.3	43	90.5	57.2	84.3
	GBDT	80.3	55.7	83.9	50.5	86.5
	KNN	65.4	33.3	89.4	48.2	81.9
	LR	79.1	44.2	90.3	57.4	84.5
Boruta	DNN	81.6	48.7	90.9	65.9	83.2
	SVM	79.1	37.1	93.6	67.6	80.6
	RF	80.5	46.6	89.8	62.2	82.4
	GBDT	79.3	47.1	88.5	59.6	82.3
	KNN	70.6	31.9	92.1	59.2	79
	LR	79.3	42.4	91.9	65.3	81.6
External validation						
LASSO regression	DNN	82.8	98.1	23.7	79.2	80.7
	SVM	72.1	47.4	78	38.9	21.6
	RF	78.6	44	75.6	34.8	21.1
	GBDT	79.6	9.5	63.2	43.3	19.1
	KNN	60.1	9	75.9	52.6	22
	LR	82.4	6.4	68.6	37.7	19.8
Boruta	DNN	75.3	94.5	25.7	79	61.1
	SVM	69.8	73.3	81.3	53.7	22.8
	RF	71.4	5.8	82.2	49.5	22.7
	GBDT	71.8	89.1	74.2	50.6	21.6
	KNN	59.6	10.4	78.6	59	22.8
	LR	74	6	73.2	39.8	20.8

*DNN* deep neural network, *SVM* supervector machine, *RF* random forest, *GDBT* gradient booster decision tree, *KNN* k-nearest neighbor, *LR* logistic regression.

**Figure 3:**
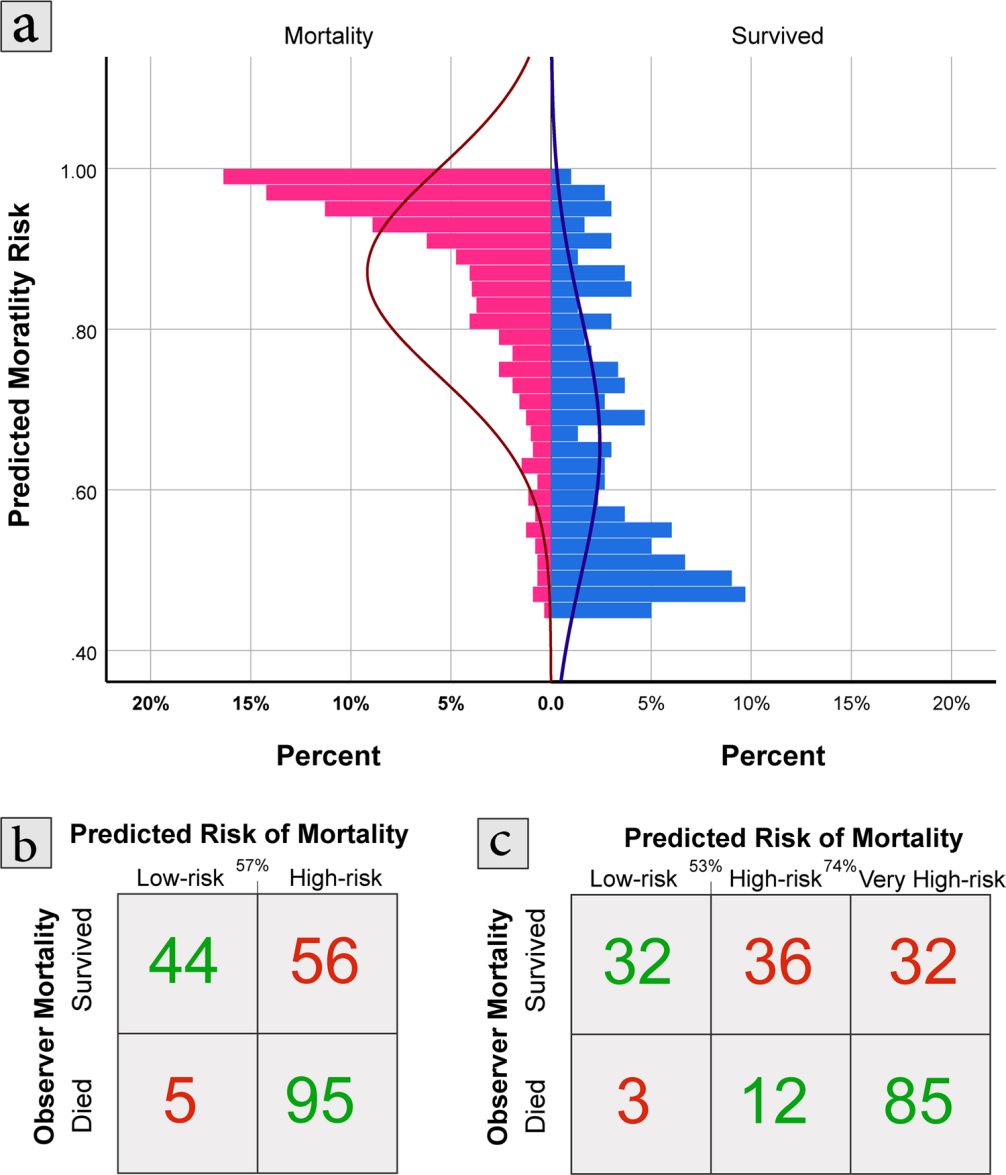
Probability graph and risk of mortality (**a**), binary confusion matrix (**b**), and ternary confusion matrix (**c**) of external validation dataset using cutoff scores suggested by clinicians.

The multivariate imputation showed a promising performance on the primary test set when 2 out of 10 laboratory variables were missing. The change in model performance ranged from -1.4% (GBDT with LASSO features) to 4.2% (KNN with LASSO variables), and the performance of the DNN model with LASSO features decreased by 1.6% when imputing two missing laboratories. The generalized performance of the DNN model using LASSO variables was confirmed in the external validation (83.4–82.8%), and the model performance change ranged between 0.7% increase (GDBT with LASSO features) to 11.9% decrease (SVM with Brouta features) in AUC. The confusion matrix of the proposed model (DNN using LASSO features) in the external validation dataset is presented in Fig. 3 using binary and ternary classification (using cutoff points offered by an expert clinician).

## Discussion

As of March 2022, different strains of the SARS-CoV-2 virus have caused five global surges in the number of cases and deaths from COVID-19. It is critical to potentiate the health system struggling with managing the resources during disease surge. The high capabilities of AI and ML algorithms in information processing can help us improve patient management. In this study, we worked intimately with healthcare professionals to provide a tool that can solve real-world needs. We developed a model to predict the mortality risk of COVID-19 inpatients at admission using clinical and laboratory data. In addition, a set of 27 clinical and ten affordable, widely available laboratories was selected in our model. Furthermore, an imputation tool is used to impute the missing labs, and a ternary outcome classification (low, high, and very high risk) was proposed as healthcare experts' suggestion.

Several studies have developed ML models to predict COVID-19 patients' mortality risk. However, as demonstrated in Table 1, models with high AUC scores are most likely trained on a small dataset or the data gathered from a single medical center. Consequently, these models may ungeneralizable, and their performance can drop in a dataset from a different center^11,14–16,18^. Furthermore, our model performed relatively better or the same as models trained on a large multicentral datasets. This higher performance may be due to the large number of input features, which can simultaneously analyze different aspects of a patient's health^10,12,13,17^.

COVID-19 can affect multiple organs, including the kidney, heart, lungs, brain, and blood. Hence, it can cause death by several different organ failures^8^. We should consider markers from several organs of the human body in order to predict the risk of mortality. Thus, as a novel approach, we collected and analyzed more than 80 on-admission features representing the function of different organs. We used a relatively large dataset to train our ML and DNN models and selected the input features using feature selection methods to eliminate collinearity. Nevertheless, overfitting of models, especially ANN, was a substantial problem in this study due to the large number of selected features for models’ input. One of the most important parameters that we added to prevent them from overfitting was L2 regularization, which resulted in a good performance in the validation dataset. Also, adding kernel regularization and 60% dropout for each layer, as well as limiting the number of neurons and hidden layers in ANN, brought about a robust and generalizable model by preventing overfitting.

We selected a DNN model trained on features determined by the LASSO regression method as our proposed model. Other studies also used LASSO method for their feature selection^12–14,24^ or prediction^23^. Despite the susceptibility of neural networks to overfitting, our DNN model performed well on the external validation due to feature selection method, large sample sizes, and layer regularization. Among 10 studies with external validation, various ML methods were used for mortality prediction, including logistic regression^15,19^, random forest^11^, regression coefficient^13^, XGBosst^14,17,18^, CatBoost^11^, neural network, and DNN^15^. Although decision tree was the most common architecture in previous studies, even largescale ones, we found higher precision for DNN. This may be due to the high number of input features and the complex interaction of predictors.

In a similar study, Gao et al. used data from 1500 patients in two centers and developed an ensembled model called MRPMC. MROMC is composed of four ML methods of logistic regression, support vector machine, gradient-boosted decision tree, and neural network^16^. However, the AUC in external validation of MRPMC, logistic regression, and neural network were fairly equal (91.8%, 91.3%, and 91.1%, respectively). Similarly, we find the neural network and logistic regression methods better for generalizable use. However, we avoided ensemble architecture to prevent overfitting since 37 input features were selected, while Gao et al. had eight. Also, ensemble models require longer prediction time, more computation power, and hard work for tuning.

The application of ML models in the clinic depends on the input features and prediction accuracy. Ease of access to input features, along with high accuracy and generalization of prediction, can increase acceptance of ML tools by healthcare workers. Selected features in the present study include 18 factors at the time of admission. Previous studies included many of our selected features for prognosis prediction, which can imply the accuracy of our feature importance method^10–12,14,15^. Laboratory markers, patient demographics, medical history, and vital signs have been used as effective features in predicting the mortality of patients with COVID-19, similar to this study^10,11,28–33^. However, we excluded some variables, such as inflammatory cytokines, while others found them predictive^34–37^. Since we excluded some features with collinearity, the other included feature represents the effect of this predictor on mortality.

The results of this study are applicable to managing COVID-19 inpatients with the current and upcoming COVID-19 surges. First, validation with 20% missing data indicates the approved potential of our model when the patient's data is unreachable and needs imputation. Second, the model's generalization was investigated using data from a fourth hospital in a different province. The AUC of 82.8% was achieved in external validation, which confirmed the model performance for global application. Third, we proposed ternary severity classification as per clinician’s opinion to show the most susceptible patients with very high severity. Our model can facilitate clinical decision-making, resource allocation, and evaluation of drug’s effectiveness by risk stratifying mortality in COVID-19 inpatients.

Nonetheless, there are some limitations to this work that should be noted. First, even though we had a relatively large patient population, our study was retrospective. Prospective validation of our study is required to ascertain the results. The hospitals in our study are all in a developing country (Iran). The scarcity of medical resources in Iranian hospitals may bring about inadequate service allocated to patients. This condition can thereby increase the mortality rate in such countries in contrast to countries with effective medical systems. Additionally, the current model does not encompass imaging, microbiological, and histological data, which could contribute to a more precise prognosis prediction despite the inconvenience. Socioeconomic and racial differences, which were investigated in some studies^38,39^, might as well play a role in prognosis.

In conclusion, this study shows that ML methods can predict the mortality risk of COVID-19 patients on admission. This approves the potential of ML methods for use in clinical practice as a decision-support system. However, effective ML models should satisfy the real-world needs of healthcare experts to increase the chance of implementation in practice. Further studies are suggested to investigate and overcome the current barriers to applying ML in medical practice.

## Supplementary Information

Supplementary Information.

## Data Availability

The datasets used in the current study are available from the corresponding author on reasonable request. The dataset would be unreservedly available for use as a validation dataset of other research projects, after sending the request to the corresponding author, or SAASN. The code related to this is available at https://github.com/SiavashShirzad/CovidAI. The code for data mining and the “Tehran COVID-19 Cohort” project information is available at  https://github.com/Sdamirsa/Tehran_COVID_Cohort. The data used in this study will be published for non-commercial use in the future at https://github.com/Sdamirsa/Tehran_COVID_Cohort.
