# Supplementary material for: Generalizable machine learning approach for COVID-19 mortality risk prediction using on-admission clinical and laboratory features
Source: Sci Rep. 2023 Feb 10;13:2399. doi: 10.1038/s41598-023-28943-z (PMC9911952; doi:10.1038/s41598-023-28943-z)

This file is supplementary to “**Generalizable Machine Learning Approach for COVID-19 Mortality Risk Prediction using On-admission Clinical and Laboratory Features**” by

Siavash Shirzadeh Barough, Seyed Amir Ahmad Safavi-Naini, Fatemeh Siavoshi, Atena Tamimi, Saba Ilkhani, Setareh Akbari, Sadaf Ezzati, Hamidreza Hatamabadi, Mohamad Amin Pourhoseingholi.

Corresponding to: Dr Mohamad Amin Pourhoseingholi, PhD, Basic and Molecular Epidemiology of Gastrointestinal Disorders Research Center, Research Institute for Gastroenterology and Liver Diseases, Shahid Beheshti University of Medical Sciences, Tehran, Iran, Pourhoseingholi@sbmu.ac.ir

Supplementary Table S1. Mean difference and characteristics of mortality and survived cohort.

Feature	Mortality Cohort (N=917)	Survived Cohort (N=4403)	P-Value
Headache	58.0(6.32%)	379.0(2.98%)	0.0001
vomiting	180.0(19.63%)	767.0(6.04%)	0.0001
HLP	44.0(4.80%)	155.0(1.22%)	0.158
Myalgia	181.0(19.74%)	895.0(7.05%)	0.0001
Cough	373.0(40.68%)	1402.0(11.04%)	0.0001
Fever	343.0(37.40%)	1312.0(10.33%)	0.0001
Opium	43.0(4.69%)	135.0(1.06%)	0.39
PH	7.36(7.29,7.41)	7.38(7.34,7.42)	0.0001
Anosmia	5.0(0.55%)	54.0(0.43%)	0.001
Ear pain	0.0(0.00%)	5.0(0.04%)	0.231
CA	8.50(8.00,9.10)	8.70(8.20,9.23)	0.0001
HB	11.80(10.00,13.30)	12.40(11.00,13.60)	0.0001
o2sat	85.00(78.00,90.00)	90.00(85.72,93.00)	0.0001
HCO3	23.70(20.20,27.40)	26.00(23.20,28.70)	0.0001
Gender	nan(0.00%)	nan(0.00%)	0.0001
Dyspnea	557.0(60.74%)	1698.0(13.37%)	0.126
rigors	218.0(23.77%)	870.0(6.85%)	0.0001
Weakness	379.0(41.33%)	1027.0(8.09%)	0.118
Sore throat	14.0(1.53%)	73.0(0.57%)	0,046
Anorexiua	143.0(15.59%)	522.0(4.11%)	0.012
Diarrhea	76.0(8.29%)	334.0(2.63%)	0.0001
Chest pain	63.0(6.87%)	319.0(2.51%)	0.0001
Seizure	12.0(1.31%)	21.0(0.17%)	0.107
Lymph adenopathy	0.0(0.00%)	3.0(0.02%)	0.416
Dermatic lesions	8.0(0.87%)	5.0(0.04%)	0.006
Abdominal pain	61.0(6.65%)	195.0(1.54%)	0.307
Hemorragia	9.0(0.98%)	11.0(0.09%)	0.044

Hemi paresia	9.0(0.98%)	20.0(0.16%)	0.303
Pregnancy	2.0(0.22%)	17.0(0.13%)	0.104
Smoker	60.0(6.54%)	160.0(1.26%)	0.275
Hookah	4.0(0.44%)	20.0(0.16%)	0.233
HTN	419.0(45.69%)	997.0(7.85%)	0.0001
CABG	65.0(7.09%)	120.0(0.95%)	0.002
Ashtma	23.0(2.51%)	87.0(0.69%)	0.164
Pneumonia	7.0(0.76%)	7.0(0.06%)	0.041
CKD	64.0(6.98%)	111.0(0.87%)	0.0001
Thyroid problems	51.0(5.56%)	156.0(1.23%)	0.439
immunodefficacy	1.0(0.11%)	6.0(0.05%)	0.436
Tuberclosis	3.0(0.33%)	10.0(0.08%)	0.572
Anemia	13.0(1.42%)	40.0(0.32%)	0.517
Liver steatosis	4.0(0.44%)	22.0(0.17%)	0.172
LYMPHH	10.00(7.00,16.40)	16.00(10.00,24.30)	0.0001
PLT	185.00(135.00,259.00)	192.00(149.00,255.00)	0.0001
AST	45.00(29.00,73.90)	36.00(25.50,53.00)	0.0001
ALT	30.00(18.00,50.38)	29.65(18.42,47.57)	0.0001
CRP	41.20(14.30,77.80)	28.50(10.80,61.10)	0.0001
pco2	42.80(36.50,50.10)	45.00(39.40,50.30)	0.0001
troponin	0.02(0.00,0.12)	0.01(0.00,0.03)	0.0001
cpk	161.00(80.00,379.00)	118.00(63.00,264.00)	0.0001
pt	13.00(12.40,14.60)	13.00(12.00,13.50)	0.0001
ptt	32.00(27.00,38.00)	31.00(26.70,35.00)	0.0001
esr	36.00(21.00,58.00)	33.24(20.00,53.00)	0.0001
NA	138.00(135.00,141.00)	138.00(135.00,140.00)	0.0001
pr	88.00(80.00,100.00)	86.00(80.00,94.00)	0.0001
DBP	75.00(70.00,80.00)	80.00(70.00,80.00)	0.0001
SBP	116.00(100.00,130.00)	120.00(110.00,130.00)	0.0001
T	85.00(78.00,90.00)	80.00(70.00,85.00)	0..520
NEUT	85.00(78.00,90.00)	80.00(70.00,85.00)	0.0001
RR	19.09(18.00,22.00)	18.00(18.00,20.00)	0.0001
Age	74.00(61.00,83.00)	60.00(47.00,71.00)	0.0001
WBC	9.20(6.30,13.30)	6.80(4.90,9.70)	0.0001
DM	346.0(37.73%)	784.0(6.17%)	0.0001
k	4.20(3.80,4.60)	4.00(3.80,4.40)	0.0001
Cancer	78.0(8.51%)	128.0(1.01%)	0.0001
inr	1.14(1.00,1.30)	1.07(1.00,1.20)	0.0001
IHD	214.0(23.34%)	394.0(3.10%)	0.0001
CR	1.40(1.10,2.20)	1.10(0.90,1.40)	0.0001
CHF	31.0(3.38%)	52.0(0.41%)	0.01
COPD	22.0(2.40%)	47.0(0.37%)	0.133
CVA	101.0(11.01%)	134.0(1.06%)	0.0001
GI problems	15.0(1.64%)	35.0(0.28%)	0.271
Hepatitis C	1.0(0.11%)	4.0(0.03%)	0.625

Alzheimer	63.0(6.87%)	48.0(0.38%)	0.0001
Arthralgia	14.0(1.53%)	40.0(0.32%)	0.515
Psychological problems	24.0(2.62%)	39.0(0.31%)	0.017
Rheumatoid Arthritis	13.0(1.42%)	27.0(0.21%)	0.193
mg	2.00(1.80,2.20)	1.90(1.80,2.10)	0.0001
rhinorrhea	9.0(0.98%)	20.0(0.16%)	0.303
Parkinson	25.0(2.73%)	24.0(0.19%)	0.0001
Loss of consciousness	233.0(25.41%)	179.0(1.41%)	0.0001
Alcohol consumption	10.0(1.09%)	11.0(0.09%)	0.022

Supplementary Table S2. Cox regression of variables for mortality incidence.

Feature	HR	Lower 95% CI	Upper 95% CI	P-Value
Headache	0.881	0.668	1.164	0.374
vomiting	0.830	0.696	0.990	0.038
HLP	0.957	0.697	1.315	0.788
Myalgia	0.825	0.688	0.988	0.037
Cough	0.946	0.811	1.104	0.481
Fever	0.936	0.774	1.133	0.500
Opium	0.827	0.581	1.178	0.293
PH	0.651	0.413	1.024	0.063
Anosmia	0.925	0.380	2.253	0.864
Ear pain	0.000	0.000	########	0.904
CA	0.979	0.919	1.042	0.501
HB	0.962	0.931	0.995	0.025
o2sat	0.970	0.965	0.976	0.000
HCO3	0.971	0.957	0.986	0.000
Gender	0.991	0.853	1.151	0.906
Dyspnea	0.925	0.792	1.081	0.326
rigors	0.978	0.789	1.213	0.842
Weakness	1.025	0.889	1.183	0.731
Sore throat	0.828	0.481	1.426	0.496
Anorexia	0.846	0.698	1.026	0.090
Diarrhea	1.180	0.922	1.509	0.189
Chest pain	0.877	0.668	1.152	0.346
Seizure	0.973	0.501	1.888	0.935
Lymph adenopathy	0.001	0.000	########	0.926
Dermatic lesions	3.221	1.537	6.750	0.002
Abdominal pain	1.063	0.803	1.408	0.669
Hemorragia	0.761	0.380	1.521	0.439
Hemi paresia	1.161	0.586	2.299	0.669
Pregnancy	0.960	0.122	7.552	0.969
Smoker	0.860	0.631	1.171	0.338
Hookah	0.578	0.162	2.055	0.397
HTN	0.890	0.766	1.035	0.132
CABG	1.184	0.899	1.560	0.229
Asthma	0.800	0.518	1.233	0.312
Pneumonia	1.243	0.556	2.776	0.597
CKD	0.775	0.576	1.044	0.094
Thyroid problems	1.195	0.887	1.610	0.241
Immune defficacy	1.619	0.221	11.854	0.635
Tuberculosis	0.913	0.276	3.020	0.881
Anemia	1.222	0.685	2.182	0.497
Liver steatosis	1.249	0.460	3.390	0.663
LYMPHH	1.005	0.987	1.024	0.576

PLT	0.999	0.998	1.000	0.002
AST	1.000	1.000	1.001	0.804
ALT	1.000	0.999	1.001	0.811
CRP	1.002	1.000	1.003	0.039
pco2	1.004	0.999	1.008	0.084
troponin	1.011	0.987	1.035	0.368
CPK	1.000	1.000	1.000	0.006
pt	1.000	0.997	1.004	0.848
ptt	1.002	0.995	1.008	0.619
esr	0.999	0.996	1.003	0.715
NA	1.003	0.991	1.015	0.611
pr	1.005	1.002	1.009	0.004
DBP	1.000	0.999	1.001	0.740
SBP	0.999	0.996	1.002	0.537
T	1.000	0.995	1.005	0.972
NEUT	1.019	1.003	1.036	0.019
RR	1.009	1.002	1.016	0.016
Age	1.028	1.023	1.034	0.000
WBC	1.008	1.002	1.015	0.015
DM	1.090	0.936	1.270	0.266
k	1.040	0.991	1.091	0.111
Cancer	1.253	0.966	1.626	0.089
INR	1.100	0.954	1.267	0.188
IHD	1.101	0.927	1.309	0.272
CR	1.041	1.000	1.085	0.051
CHF	1.129	0.761	1.675	0.546
COPD	1.181	0.755	1.849	0.466
CVA	1.207	0.957	1.522	0.112
GI problems	1.797	1.037	3.113	0.037
Hepatitis C	1.348	0.185	9.805	0.768
Alzheimer	1.038	0.776	1.387	0.802
Arthralgia	0.992	0.555	1.775	0.979
Psychological problems	1.636	1.073	2.495	0.022
Rheumatoid Arthritis	1.908	1.062	3.427	0.031
mg	1.020	0.836	1.243	0.848
rhinorrhea	1.892	0.926	3.868	0.080
Parkinson	1.106	0.720	1.700	0.645
Loss of consciousness	1.499	1.253	1.794	0.000
Alcohol consumption	2.599	1.235	5.469	0.012

Supplementary Figure S1. Feature selection by Boruta method.

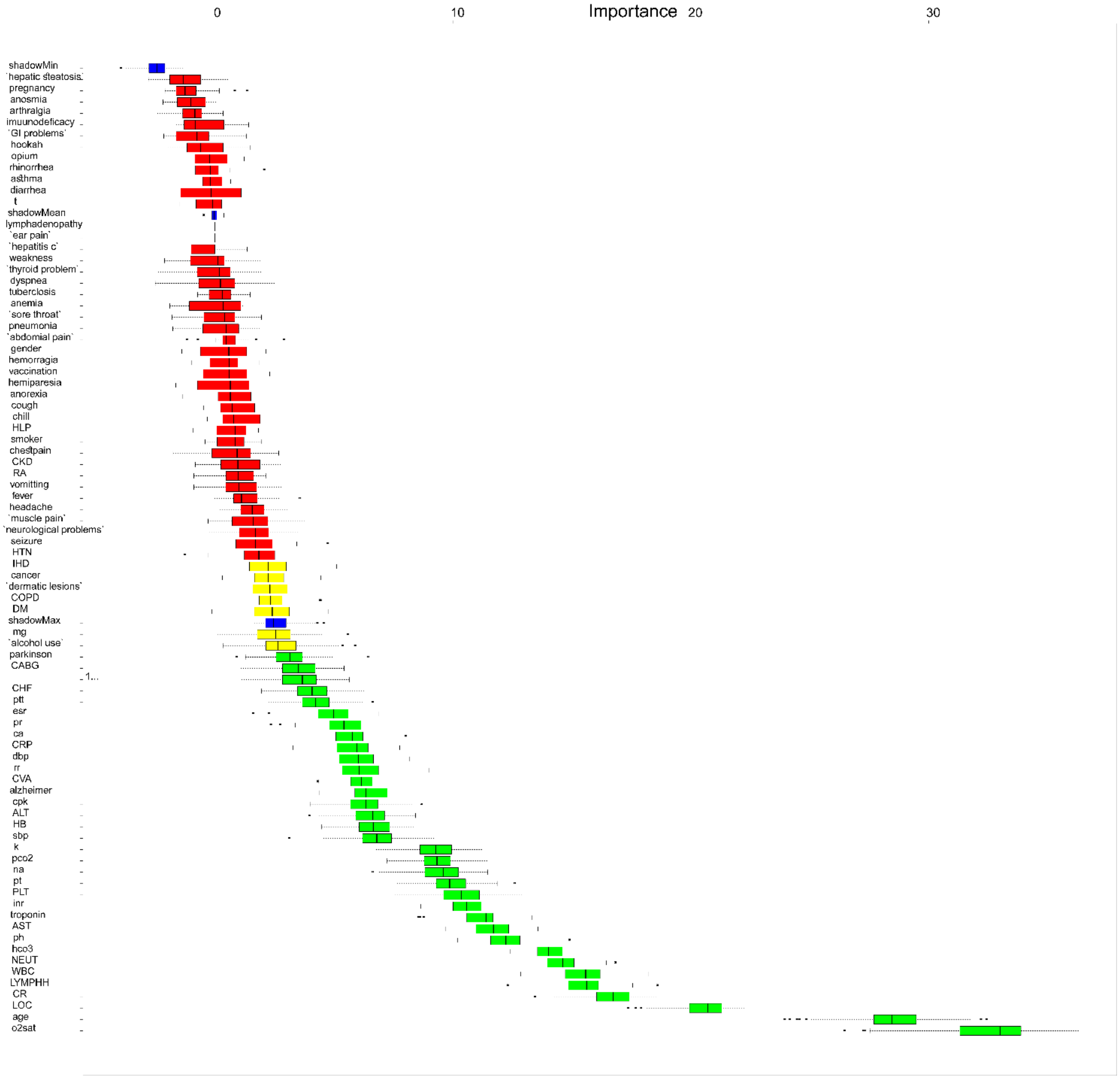

Supplementary Figure S2. Feature Selection by LASSO method.

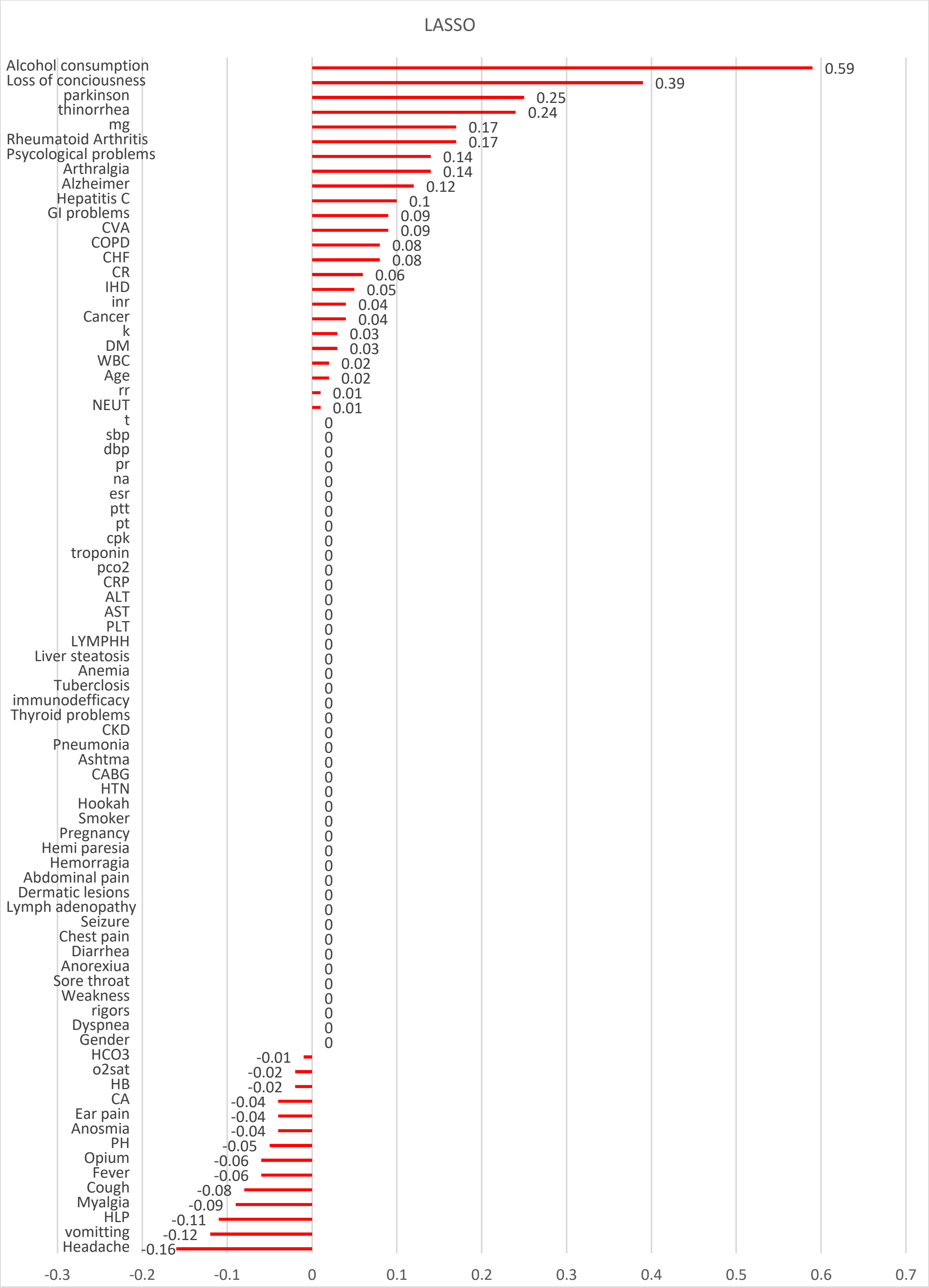

Supplementary Figure S3. Correlation gram of nominal variables.

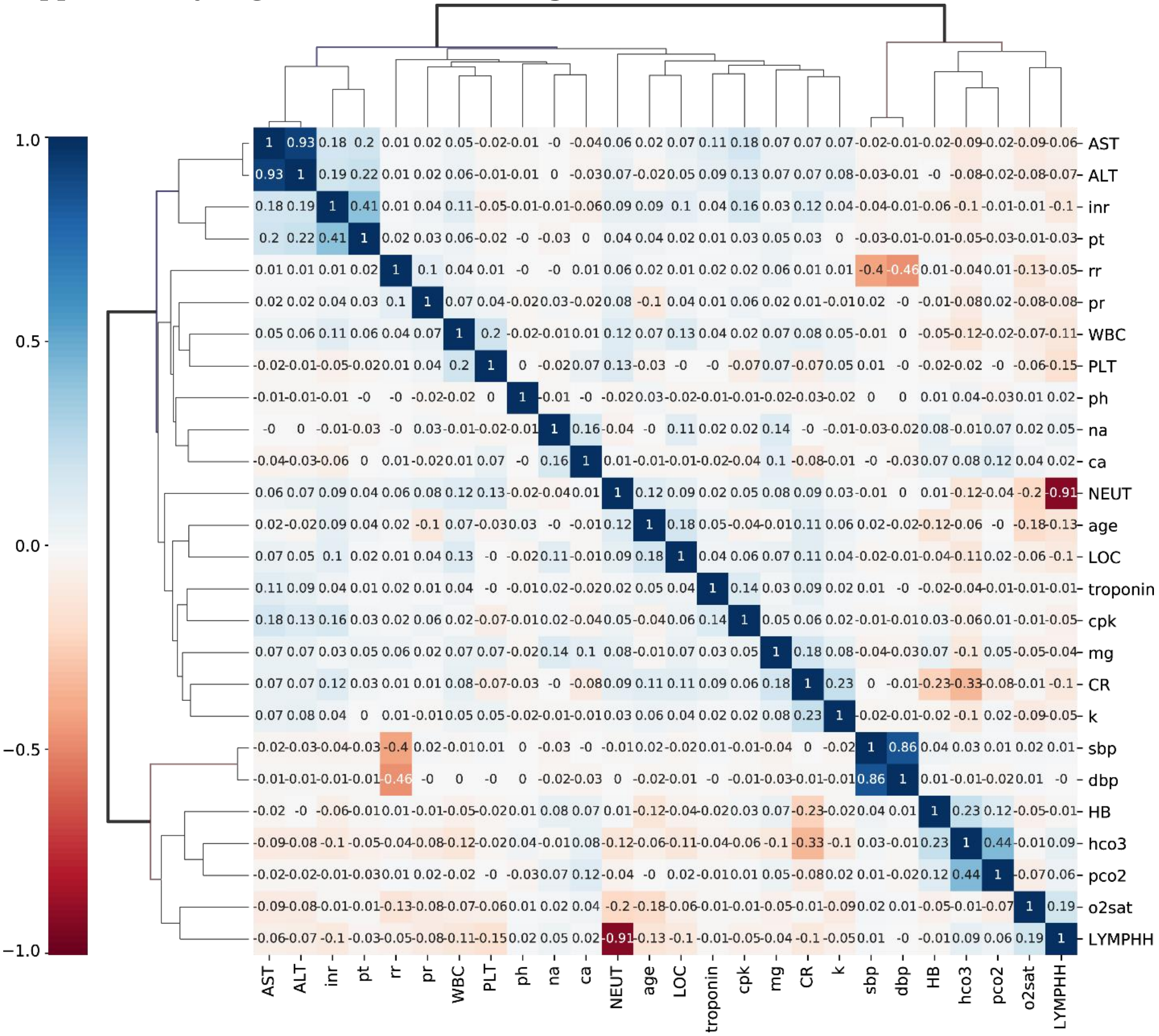

Supplement: Supplementary file 1 — Supplementary Information. [file 41598_2023_28943_MOESM1_ESM.pdf]
